# Functional characterization of *GmBZL2* (*AtBZR1* like gene) reveals the conserved BR signaling regulation in *Glycine max*

**DOI:** 10.1038/srep31134

**Published:** 2016-08-08

**Authors:** Yu Zhang, Yan-Jie Zhang, Bao-Jun Yang, Xian-Xian Yu, Dun Wang, Song-Hao Zu, Hong-Wei Xue, Wen-Hui Lin

**Affiliations:** 1 State Key Laboratory of Plant Molecular Physiology, Institute of Botany, Chinese Academy of Sciences, Beijing, China; 2 University of Chinese Academy of Sciences, Beijing, China; 3 School of Life Sciences and Biotechnology, Shanghai Jiao Tong University, Shanghai, China; 4 National Key Laboratory of Plant Molecular Genetics, Institute of Plant Physiology & Ecology, Shanghai Institutes for Biological Sciences, Chinese Academy of Sciences, Shanghai, China; 5 State Key Laboratory of Systematic and Evolutionary Botany, Institute of Botany, Chinese Academy of Sciences, Beijing, China; 6 Department of Plant and Microbial Biology, University of California, Berkeley, CA, USA

## Abstract

Brassinosteroids (BRs) play key roles in plant growth and development, and regulate various agricultural traits. Enhanced BR signaling leads to increased seed number and yield in Arabidopsis *bzr1-1D* (AtBZR1^P234L^, gain-of-function mutant of the important transcription factor in BR signaling/effects). BR signal transduction pathway is well elucidated in Arabidopsis but less known in other species. Soybean is an important dicot crop producing edible oil and protein. Phylogenetic analysis reveals *AtBZR1*-like genes are highly conserved in angiosperm and there are 4 orthologues in soybean (*GmBZL1-4*). We here report the functional characterization of *GmBZL2* (relatively highly expresses in flowers). The P234 site in AtBZR1 is conserved in GmBZL2 (P216) and mutation of GmBZL2^P216L^ leads to GmBZL2 accumulation. GmBZL2^P216L^ (GmBZL2*) in Arabidopsis results in enhanced BR signaling; including increased seed number per silique. GmBZL2* partially rescued the defects of *bri1-5*, further demonstrating the conserved function of GmBZL2 with AtBZR1. BR treatment promotes the accumulation, nuclear localization and dephosphorylation/phosphorylation ratio of GmBZL2, revealing that GmBZL2 activity is regulated conservatively by BR signaling. Our studies not only indicate the conserved regulatory mechanism of GmBZL2 and BR signaling pathway in soybean, but also suggest the potential application of GmBZL2 in soybean seed yield.

BR plays important roles in plants growth and development, such as cell elongation, division and differentiation, seed germination, photomorphogenesis, reproductive development, plant immunity, stomatal development, and so on^1^,^2^,^3^,^4^,^5^,^6^. In *Arabidopsis thaliana,* many BR-deficient and insensitive mutants like *det2*, *cpd*, *dwf4*, *bri1*, *bin2*, have been identified, and most of them show light-grown morphology in dark and dwarfism phenotype in light^4^.

Molecular and genetic studies have illustrated a clear BR signal transduction pathway in Arabidopsis. When BR level is low, the BR receptor BRASSINOSTEROID INSENSITIVE1 (BRI1, a cell-surface receptor kinase) is inactive, the protein BRI1 KINASE INHIBITOR1 (BKI1) binds to BRI1 and inhibits BRI1. A GSK3-like kinase, BRASSINOSTEROID INSENSITIVE2 (BIN2), is phosphorylated and activated, which can phosphorylate two homologous transcription factors BRASSINAZOLE RESISTANT1 (BZR1) and BZR2 [also named BRI1-EMS-SUPPRESSOR1 (BES1)]^7^,^8^,^9^. Phosphorylated BZR1 and BZR2 cannot target DNA in the nuclear and are restrained in the cytoplasm by 14-3-3 protein, and degraded by proteasome system^10^,^11^,^12^.

As BR level increasing, BR directly binds to the extracellular domain of BRI1 and triggers the whole transduction cascade^13^,^14^,^15^,^16^. BRI1 recruits the co-receptor BRI1-ASSOCIATED RECEPTOR KINASE1 (BAK1) to sequential trans-phosphorylated with each other, and at the same time the inhibitory protein BKI1 is phosphorylated and disassociates from BRI1^17^,^18^,^19^. BR activation leads to BRI1 phosphorylate BRASSINOSTEROID-SIGNALLING KINASE1 (BSK1) and CONSTITUTIVE DIFFERENTIAL GROWTH1 (CDG1); both of them are plasma membrane-anchored cytoplasmic kinases^20^,^21^. Then BSK1 and CDG1 phosphorylate the phosphatase BRI1-SUPPRESSOR1 (BSU1)^20^,^22^,^23^. BSU1 dephosphorylates BIN2 through a conserved tyrosine residue to deactivate BIN2 and pass it to proteasome system for degradation^14^,^24^.

Thus, dephosphorylated BIN2 loses its ability to phosphorylate BZR1 and BZR2. In the meantime, PROTEIN PHOSPHATASE 2A (PP2A) can dephosphorylate BZR1 and BZR2^25^, which helps their actions in nuclear. Then activated BZR1 and BZR2 accumulate in nuclear and binds to DNA sequences of their target genes (mostly in special elements of promoters) to induce plant BR responses^26^,^27^,^28^,^29^. Besides the main pathway, BKI1, which releases from BRI1, binds to 14-3-3 protein to prevent its function, the action which also enhances BZRs activity and BR signaling^18^.

Our publications reported previously that BR signaling positively regulated the seed number per silique and seed size in Arabidopsis^30^,^31^. The seed number increases in *bzr1-1D*; while decreases severely in BR insensitive mutant *bri1-5* and BR deficient mutant *det2*^30^. Furthermore, the seed size/weight and silique number do not show significantly difference in *bzr1-1D*, which means the increasing seed number per silique enhances the final seed yield^31^.

Although BR signaling pathway is well studied in Arabidopsis, the regulation mechanisms in dicot crops, such as soybean (*Glycine max*) and *Brassica napus*, remains poorly understood. Seed yield is a critical trait for these crops. Soybean, one of the important dicot crops, provides not only edible oil but also protein for human life all around the world^32^. For satisfying the worldwide demand for food, elevating the soybean yield is urgent. Seed yield depends on seed weight and seed number^33^,^34^. Increasing the pod number and seed number per pod are two key ways to improve the seed yield^34^. In the past decades, researches of seed development mainly focused on the seed size and weight, because people considered that increasing seed number would reduce seed weight and quality because of limited space and nutrition. There is no systemic research of the seed number regulation (mainly in monocot crop). However, currently several reports reveal that increasing seed number without obvious seed weight loss can improve the seed yield significantly^35^,^36^. Besides, it is well-known that the external environmental factors and internal hormonal signalings cooperate to regulate the reproductive progress including the seed number and size^37^,^38^. The phytohormone brassinosteroid (BR) also will be a key player in seed development and yield^30^,^31^.

In this study, using BLASTP tools to search soybean proteome sequences with the full-lengh AtBZR1 protein sequence we identified four orthologues of *AtBZR1* in soybean, which named *GmBZL1-4* (*Glycine max AtBZR1 like gene 1-4*) and focused on *GmBZL2*. We overexpressed *GmBZL2* by point mutation conserved with *AtBZR1* in Arabidopsis wild type Col-0 and BR mutant to investigate *GmBZL2* functions in plant growth and development, as well as seed number regulation. The results demonstrate conservation of BR signaling regulation in dicot species, which gives potential application to increase seed yield of dicot crops.

## Results

### Phylogenetic relationships of *AtBZR1* like genes

**Figure 1:**
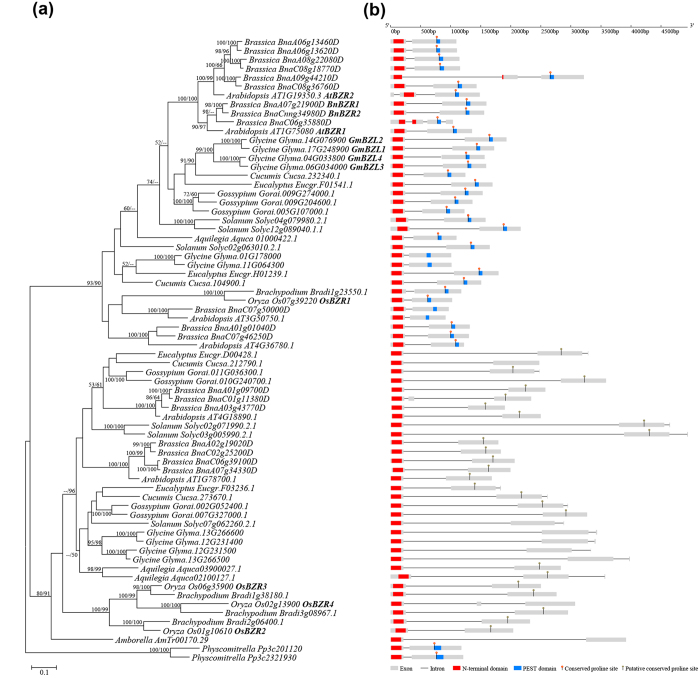
Phylogenetic relationships and exon-intron structures of *BZR1*-like genes. (**a**) Phylogenetic tree of *BZR1*-like genes. The bootstrap values (>50%) of Maximum likelihood and Neighbor-Joining analyses are shown next to the nodes. (**b**) Schematic representation of exon-intron structures. Exons and introns are represented by boxes and lines, respectively. The N-terminal domain and PEST domain are indicated with red and blue boxes, respectively. The conserved proline site (P234L mutated in *bzr1-1D*) in the PEST domain is represented by an orange matchstick, and the putative conserved proline site that is homologous to that in PEST domain is indicated with a gray matchstick.

The phylogenetic tree shows that angiosperm *BZR1*-like genes form two groups, which was strongly supported by both ML and NJ methods. To facilitate description, we named the two groups as *BZR1* and *BZR2/3/4* according to homologous genes in rice. Genes from core eudicots, basal eudicots, and monocots can be found in both *BZR1* and *BZR2/3/4* groups, but only one *BZR1*-like gene (*Amborella AmTr00170.29*) was identified in the basal most extant angiosperm, *Amborella trichopoda*, which belongs to the *BZR2/3/4* group (Fig. 1a). The results suggest that the groups *BZR1* and *BZR2/3/4* may be generated by the gene duplication event before the diversification of extant angiosperms, but later *A. trichopoda* lost the *BZR1* member during the evolution.

Based on the exon-intron structure analysis, all the members in the *BZR1* group contain two exons other than *AT1G19350.3* (*AtBZR2*, 3 exons) in Arabidopsis, *BnaA09g44210D* (3 exons) and *BnaC06g35880D* (4 exons) in Brassica (Fig. 1a). It is possible that ancestral *BZR1* gene in the most recent common ancestor (MRCA) of the angiosperm has two exons, while the exon numbers of some members of Arabidopsis and Brassica increased by 3-4 due to independent intron gain events. Compared to the *BZR1* group, most *BZR2/3/4* members have 2-3 exons (Fig. 1b). In the phylogenetic context, we inferred that the ancestral *BZR2/3/4* gene in the MRCA of ext ant angiosperms might have 2 exons. Due to independent intron gains and/or losses during the evolution, the exon number of exons differed from each other.

From the protein domain composition, we found a conserved domain at the N-terminal end which was shared by the two groups, but another conserved PEST domain was only identified in members of the *BZR1* group (Fig. 1b). It has been reported that the 234^th^ position proline residue (P234) has important function. AtBZR1 will accumulate and keep on activation when P234 mutates to leucine in gain-of-function mutant *bzr1-1D*^8^. But this site has been lost or replaced by other residues in some members of the *BZR1* group, such as Glyma.01G178000 and Glyma.11G064300 in soybean, AT3G50750.1 in Arabidopsis, and BnaC07g50000D in oilseed (Fig. 1b). Interestingly, a site showing homologous to the conserved residue (putative conserved residue P234 in AtBZR1) also can be identified in most members of the *BZR2/3/4* group, although no PEST domain was detected in this group (Fig. 1b).

In *Glycine max*, *GmBZL1* (*Glyma.17G248900*), *GmBZL2* (*Glyma.14G076900*), *GmBZL3* (*Glyma.06G034000*) and *GmBZL4* (*Glyma.04G033800*) all belong to the *BZR1* group, containing conserved residue sites in PEST domain (Fig. 1a,b). These four genes were clustered into a single branch in the phylogenetic tree and divided into two subgroups internally, suggesting that they may came from two specific duplication events in soybean evolution. Besides these four genes, we also found the other six *BZR1*-like genes in the genome of soybean, two of which (*Glyma.01G178000* and *Glyma.11G064300*) belong to *BZR1* branch, and another four (*Glyma.13G266600*, *Glyma.12G231400*, *Glyma.12G231500* and *Glyma.13G266500*) belong to *BZR2/3/4* branch (Fig. 1a).

We also selected the amino acid sequences of *BZR1*-like genes from *Glycine max* and Arabidopsis for further analysis. Apart from AtBZR2, the protein sequences of other four genes in Arabidopsis which named AtBEH1 (BES1/BZR1 HOMOLOG 1, AT3G50750), AtBEH2 (BES1/BZR1 HOMOLOG 2, AT4G36780), AtBEH3 (BES1/BZR1 HOMOLOG 3, AT4G18890), AtBEH4 (BES1/BZR1 HOMOLOG 4, AT1G78700), also share identity with AtBZR1 in Arabidopsis. However, they are located on the other branch of phylogenetic tree (Supplementary Fig. S1a), and the amino acid number of these four genes is less than AtBZR1/2 and GmBZL1/2/3/4 (Supplementary Fig. S1b). In AtBEH1/2/3/4, the amino acids of PEST domain are not conservative with other *BZR1*-like genes, which imply a functional diversion of these four genes during the evolution (Supplementary Fig. S1c).

### Identification and expression pattern of GmBZL2

**Figure 2:**
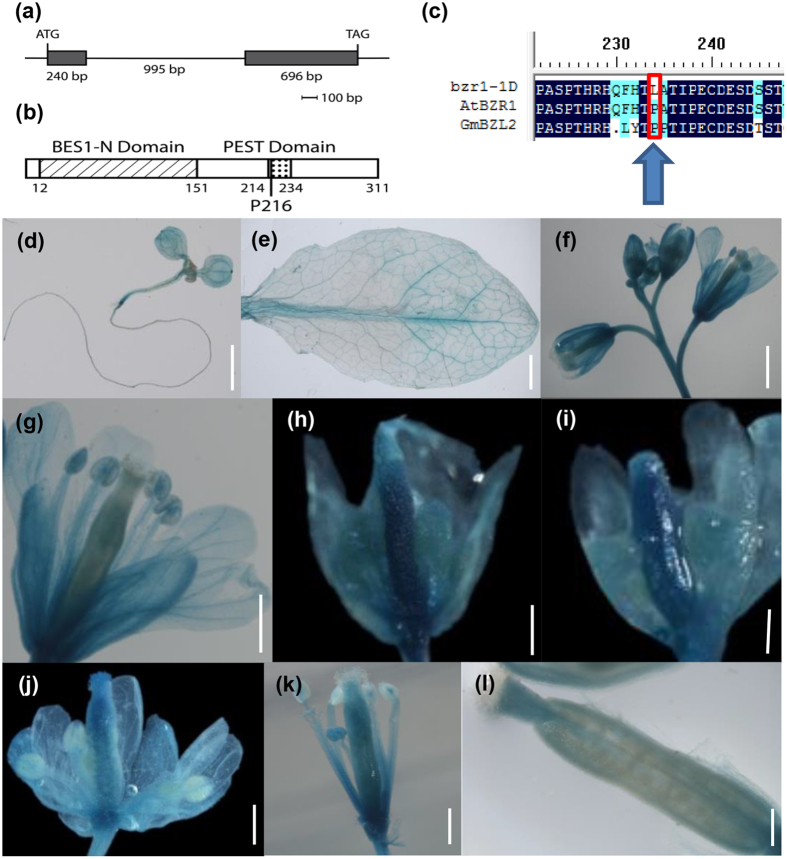
The structures of GmBZL2 and tissue specific expression pattern in Arabidopsis. (**a**) A schematic diagram of *GmBZL2* with two exons and an intron, which are represented with black boxes and intervening line. The start codon ATG starts from the +1 nucleotide, and the stop codon TAG stops at +1931 nucleotide. (**b**) The conserved domain of GmBZL2. There are two domains in the GmBZL2 sequence. BES1-N domain including 130 amino acids shares high similarity with N-terminal of AtBES1. The PEST domain contains the deduced conserved proline site P216 similar to that of AtBZR1. (**c**) The multiple alignment of the amino acid sequences in the PEST domain of *bzr1-1D*, BZR1, GmBZL2. The conserved proline residue was shown in red rectangle. (**d**) The GUS staining of 7-day-old seedling in GmBZL2-GUS transgenic line, bar = 2 mm; (**e**) rosette leaf, bar = 2 mm; (**f**) inflorescence, bar = 1 mm; (**g**) open flower, bar = 0.5 mm; (**h–k**) pistils from early stage to late stage, bars = 0.5 mm; (**l**) the silique after fertilization, bar = 0.5 mm.

Compared to other three homologous genes on Phytozome (https://phytozome.jgi.doe.gov/pz/portal.html), we found that *GmBZL2* (*Glyma14g08320.1*) had a relatively high expression level in the flowers (Supplementary Fig. S2). We speculated *GmBZL2* might have a function in the reproductive development and seed yield control. We cloned full length of *GmBZL2* from genomic DNA of Zhongdou 32 cultivar, as well as full CDS of *GmBZL2* from flower cDNA of Zhongdou 32. We found the gene structure is as same as the predicted structure. In the full genomic sequence (1931 bp), *GmBZL2* contains two exons (1-240, 1236-1931) and one intron (241-1235) (Fig. 2a). GmBZL2 shows 64.99% amino acid sequence similarity compared with AtBZR1. It also contains a conserved PEST domain (region rich in proline, glutamate, serine, and threonine) and the important proline residue (P216, conserved with P234 in AtBZR1) (Fig. 2b,c).

To confirm the expression pattern of *GmBZL2*, we generated pGmBZL2::GmBZL2-GUS Arabidopsis transgenic lines named GmBZL2-GUS. Through GUS stain assay, we found that *GmBZL2* expressed in many tissues, including leaves and flowers, especially in the early pistil and ovule (Fig. 2d–l), which indicates that the ovule development and seed number per silique might be affected by *GmBZL2*, like *AtBZR1*.

### Point mutation in conserved proline residue site enhanced GmBZL2 activity

**Figure 3:**
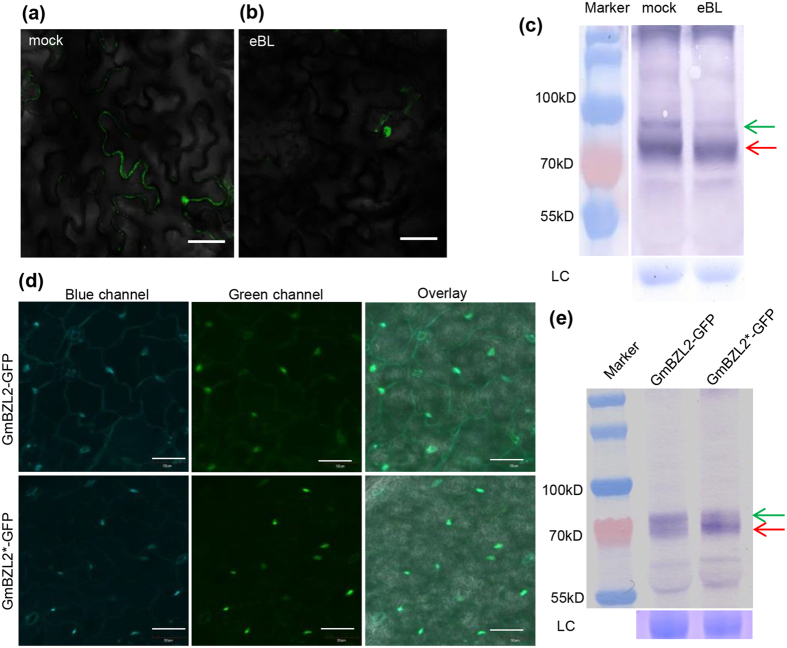
GmBZL2 has the conservative function in BR signaling pathway in Arabidopsis. (**a**,**b**) The tobacco epidermal cell expressing 35S::GmBZL2-GFP without BL treatment and with 8 hr BL treatment, bars = 50 μm. (**c**) The dephosphorylated GmBZL2 increased after 10 hr BL treatment with Western blot detection, ribosome bands were used as loading control. The green and red arrows indicate the phosphorylated and dephosphorylated GmBZL2. The five protein marker belts are 170 kD, 130 kD, 100 kD, 70 kD and 55 kD from top to bottom, LC indicates loading control. (**d**) Confocal images of cotyledon epidermal cells from 7-day-old seedling of GmBZL2-GFP and GmBZL2*-GFP transgenic lines. The blue and green channels correspond to the nuclear-associated DAPI fluorescence and GmBZL2-GFP (GmBZL2*-GFP) fluorescence, respectively. Overlay shows both channels in images. Bars = 50 μm. (**e**) The ratio of dephosphorylated/phosphorylated GmBZL2 is higher in GmBZL2*-GFP than in GmBZL2-GFP lines, ribosome bands were used as loading control. The green and red arrows indicate the phosphorylated and dephosphorylated proteins, respectively. The five protein marker belts are 170 kD, 130 kD, 100 kD, 70 kD and 55 kD from top to bottom, LC indicates loading control.

As a transcription factor, the nuclear localization of AtBZR1 is requisite for its function in Arabidopsis. The rapidly increasing of nuclear localization of AtBZR1 protein by BR treatment can be detected in transient expression system (tobacco leaves)^11^. To clarify whether BR positively regulates the localization of GmBZL2, we constructed 35S::GmBZL2-GFP vector (GmBZL2-GFP driven by the CaMV 35S promoter) and transiently expressed GmBZL2-GFP in tobacco leaves to observe the re-localization of GmBZL2 using confocal microscopy. Without BR, GmBZL2 protein located in both nuclear and cytoplasm (Fig. 3a). In contrast, most of GmBZL2 protein located in nuclear with 8 hr BR treatment (Fig. 3b). This result illustrates that BR induced nuclear localization of GmBZL2, similar to Arabidopsis BZR1.

Previous study shows that AtBZR1 is phosphorylated by BIN2 and loses its activity^7^. BR response is activated by the accumulation of dephosphorylated protein of BZR1 through BR dephosphorylating BIN2 in Arabidopsis. To investigate whether BR promotes the dephosphorylation of GmBZL2, we investigated the dephosphorylation/phosphorylation ratio of GmBZL2 in transient expression system. With 10 hr BR treatment, we extracted protein from tobacco leaves for analysis. The results indicate that the phosphorylation level of GmBZL2 decreased and the dephosphorylation level of GmBZL2 increased (Fig. 3c), suggesting that the ratio of dephosphorylated/phosphorylated GmBZL2 enhanced by BR treatment, like AtBZR1.

Similar results came from GmBZL2-GFP (pGmBZL2::GmBZL2-GFP) transgenic lines, DAPI and GFP signal of GmBZL2 proteins in cotyledon cells located in both nuclear and cytoplasm (Fig. 3d). However, In GmBZL2*-GFP (pGmBZL2::GmBZL2*-GFP) transgenic lines (overexpression transgenic lines with P216L point mutation in GmBZL2 protein, as same as P234L in AtBZR1), most of the DAPI and GFP signal of GmBZL2 proteins in cotyledon cells located in nuclear (Fig. 3d). These results illustrate that the conserved proline site in GmBZL2 would be as important as in AtBZR1 regarding to their re-localization, which demonstrates the conservation of BR positive regulation between GmBZL2 and AtBZR1.

We also found that the dephosphorylation level of GmBZL2 in GmBZL2*-GFP lines was higher than phosphorylation level of GmBZL2. In contrast, in GmBZL2-GFP transgenic lines, the dephosphorylation level of GmBZL2 was lower than phosphorylation level of GmBZL2 (Fig. 3e). These results further indicate that the conserved proline site in GmBZL2 would be as important to the GmBZL2 activity as AtBZR1 and it also marks the conservation between GmBZL2 and AtBZR1.

To further test whether GmBZL2 phosphorylation activity is contributed by BIN2^7^, we treated GmBZL2-GFP and GmBZL2*-GFP lines using a well-studied GSK3 kinase inhibitor, lithium^39^,^40^, which was capable of inhibiting phosphorylation of BZR1 by BIN2^41^,^42^. After 3 hr treatment with 100 mM LiCl, all hyperphosphorylated GmBZL2 and GmBZL2* bands disappeared in both GmBZL2-GFP and GmBZL2*-GFP transgenic lines (Supplementary Fig. S4a,b). Meanwhile, a small molecule, bikinin, can also enhance BR signaling through directly binding BIN2 and acting as an ATP competitor^43^. With 5 μM bikinin treatment, the phosphorylation band disappeared in GmBZL2*-GFP line (Supplementary Fig. S4c) and the ratio of dephosphorylation/phosphorylation of GmBZL2 also increased in GmBZL2-GFP line (Supplementary Fig. S4d). These results further demonstrate that BIN2 can enhance the phosphorylation level of GmBZL2, just like phosphorylating AtBZR1, and the BR signal transduction and the regulation of GmBZL2 might be conserved between soybean and Arabidopsis.

### GmBZL2* overexpression reproduces the *bzr1-1D* phenotypes

**Figure 4:**
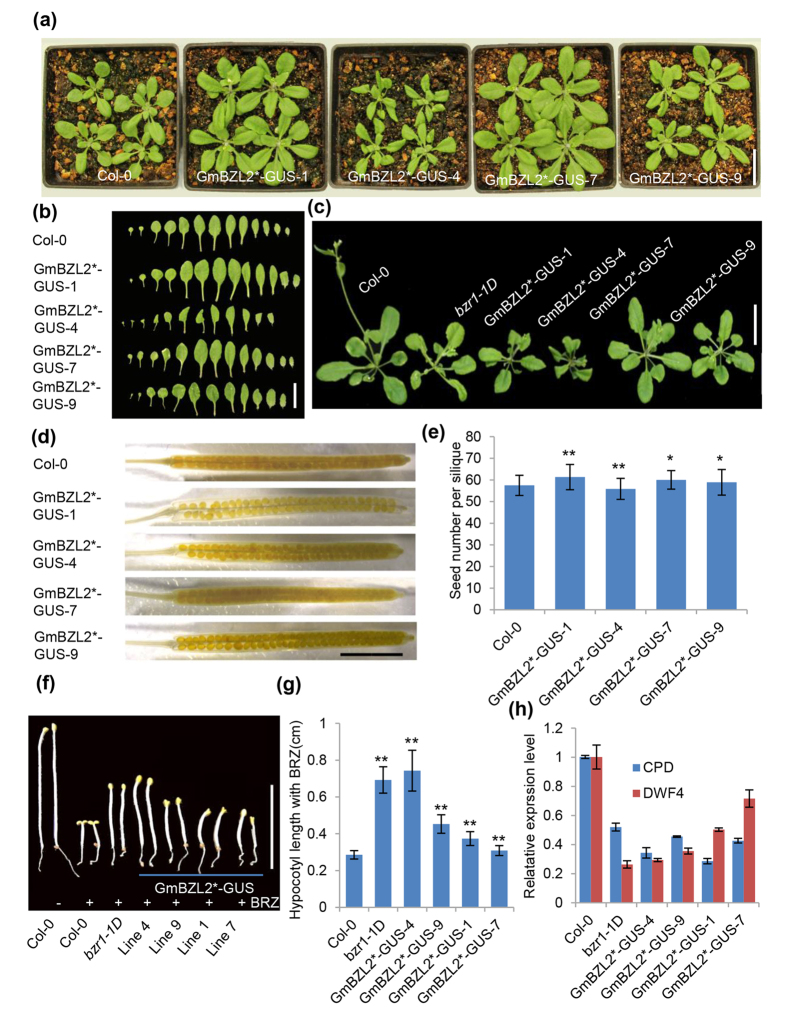
The characterization of the GmBZL2*-GUS transgenic lines. (**a**) Four-week-old plants of the Col-0, GmBZL2*-GUS transgenic lines. Bar = 2.5 cm. (**b**) The rosette leaves from the plants of Col-0 and GmBZL2*-GUS transgenic lines from the top to the bottom. Bar = 2 cm. (**c**) Six-week-old plants of the Col-0, *bzr1-1D*, and GmBZL2*-GUS transgenic lines. Bar = 2 cm. (**d**) The transparent siliques of Col-0 and GmBZL2*-GUS transgenic lines. Bar = 4 mm. (**e**) Statistical analysis of seed number per silique from the 3^rd^ to 13^th^ silique on the primary stem (a total of 5 plants from each line) of wild type and transgenic lines. The student *t* test was used to analyze the significant differences between wild type and GmBZL2*-GUS transgenic lines (*p < 0.05, **p < 0.01). (**f**) The 6-day-old seedlings of Col-0 without BRZ treatment, Col-0, *bzr1-1D*, and GmBZL2*-GUS transgenic lines grown on the 1/2MS medium with BRZ in dark. Bar = 1 cm. (**g**) Statistical analysis of the length of 6-day-old seedling hypocotyls of wild type. *bzr1-1D* and GmBZL2*-GUS transgenic lines with BRZ treatment in dark condition (a total of 15 plants from each line). The student *t* test was used to analyze the significant differences between the wild type and *bzr1-1D*, GmBZL2*-GUS transgenic lines with BRZ treatment (**p < 0.01). (**h**) The expression level of *CPD* and *DWF4* in 10-day-old seedlings of Col-0, *bzr1-1D*, and GmBZL2*-GUS transgenic lines.

BIN2 is a negative regulator of BR signaling through phosphorylating and inactivating BZR1 in Arabidopsis. BR signal-enhanced mutant *bzr1-1D* contains high dephosphorylated/phosphorylated ratio of BZR1 and shows curly leaves and delayed flowering. To confirm the function of GmBZL2, we overexpressed GmBZL2 in Arabidopsis Col-0 by introducing GmBZL2*-GUS (pGmBZL2::GmBZL2*-GUS) vector. GmBZL2*-GUS contains a conserved proline mutation site (P216L) according to the mutation site of *bzr1-1D* (P234L). All of transgenic lines showed curly leaves and delayed flowering, similar to the phenotype of *bzr1-1D* (Fig. 4a–c). In contrast, all the GmBZL2-GUS lines did not show any significant difference with wild type (Supplementary Fig. S3a), demonstrating the accumulation of dephosphorylated GmBZL2 (GmBZL2*) is the functional pattern in BR signaling in soybean, as same as Arabidopsis.

Arabidopsis seed number can be positively regulated by BR. MX3 and M4C (independent transgenic lines of pBZR1::BZR1*-CFP) have more seed number than the control line W2C (transgenic lines of pBZR1::BZR1-CFP)^30^. Our results indicate that GmBZL2*-GUS transgenic lines also had more seed number per silique than Col-0. The average seed number of Col-0 was 57.5, with different individual plants had seed set ranging from 52.8 to 62.1. The seed number of independent GmBZL2*-GUS transgenic lines was 61.3, 55.8, 60.0, 58.9 in line 1, line 4, line7, line 9 (the increase of seed number per silique is slightly), respectively (Fig. 4d,e). We infer that the less seed number in line 4 is due to a feedback inhibition by high level of BR signaling because that GUS activity was stronger in line 4 (similar in line 9) than line 1 and line 7 (Supplementary Fig. S5a–d), and the rosette leaves of line 4 (similar in line 9) are also smaller than line 1 and line 7 (Fig. 4a), indicating that too high BR signaling feedback inhibit plant growth in line 4 (detailed description in discussion). However, GmBZL2-GUS transgenic lines had similar average seed number compared with Col-0 (Supplementary Fig. S3b). These results suggest that GmBZL2* positively regulated Arabidopsis seed number, like *bzr1-1D*.

Brassinazole (BRZ) is a triazole compound which blocks BR biosynthesis specifically^44^. With BRZ treatment, wild type Arabidopsis behaves de-etiolation and dwarf phenotypes similar to BR-deficient mutants^44^. The hypocotyl length of *bzr1-1D* is longer than Col-0 with BRZ treatment in dark because enhanced BR signaling leads to BRZ insensitivity, sequential to etiolation of seedlings^8^. Compared with wild-type seedling, GmBZL2*-GUS transgenic lines had longer hypocotyl in dark (Fig. 4f,g), indicating BR signaling in GmBZL2*-GUS transgenic lines was stronger than Col-0. Corresponding to the strongest phenotype, GmBZL2*-GUS transgenic line 4 had the longest hypocotyl (Fig. 4f,g), which suggests line 4 has highest BR signaling among the transgenic lines, and shows constantly feedback phenotypes such as smaller rosette leaves and less seeds per silique.

In light, Arabidopsis BZR1 plays a role in feedback inhibition of BR biosynthesis and growth responses, resulting in shorter hypocotyls of *bzr1-1D*^26^. On the other hand, *bzr1-1D* is hypersensitive to BR with longer hypocotyls after BR treatment^26^.We found the hypocotyl lengths of *bzr1-1D* and GmBZL2*-GUS transgenic lines were slightly shorter than that of the wild type on 1/2MS medium (Supplementary Fig. S6a), while the hypocotyl length increased significantly with 10 nM eBL treatment, especially in line 4. This shows GmBZL2* is also hypersensitive to BR.

BZR1 directly suppresses the expression levels of the BR biosynthesis genes *CPD* and *DWF4 in vivo* by feedback inhibition. In *bzr1-1D* mutant the expression levels of *CPD* and *DWF4* are both down-regulated^8^,^26^. To confirm the enhanced BR signaling in GmBZL2*-GUS transgenic lines, we detected the expression level of *CPD* and *DWF4* in 10-day-old seedling using qRT-PCR, and found that the expression level of *CPD* and *DWF4* were all down-regulated in transgenic lines compared to Col-0 (Fig. 4h). This result illustrates that the dephosphorylated GmBZL2* can enhance the BR signaling and cause the same phenotype of *bzr1-1D*^26^.

**Figure 5:**
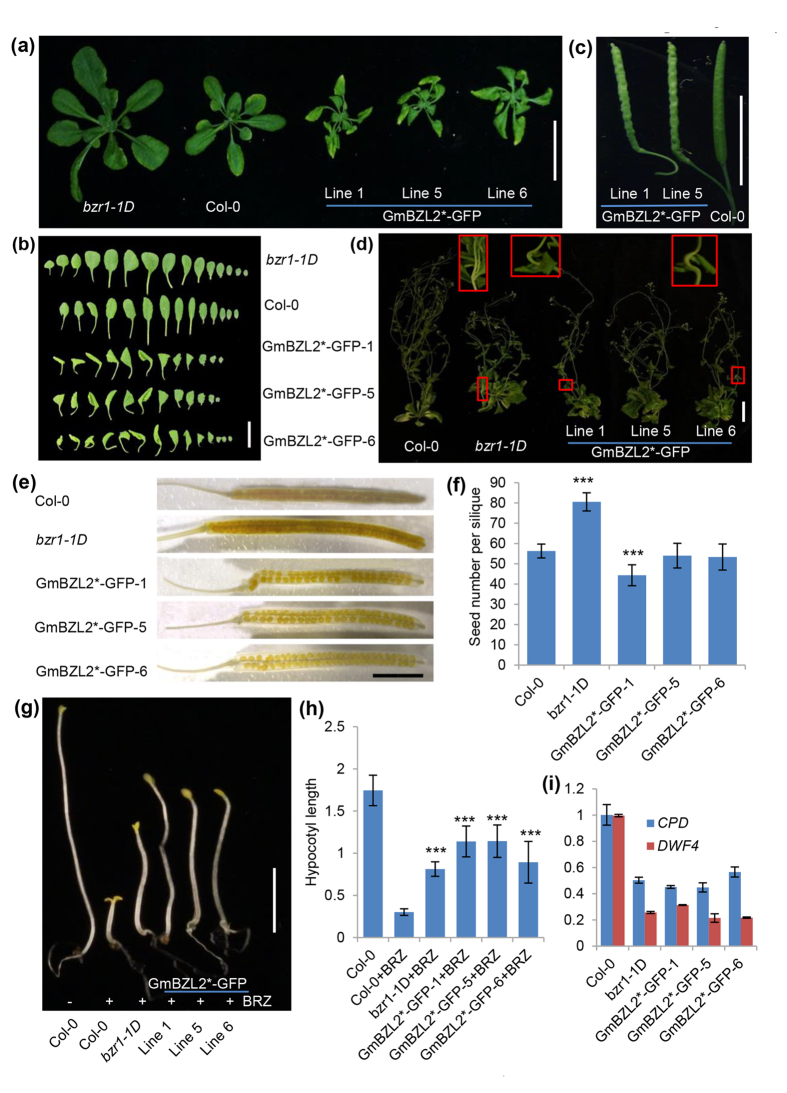
The characterization of the GmBZL2*-GFP transgenic lines. (**a**) The 4-week-old plants of *bzr1-1D*, Col-0 and the GmBZL2*-GFP lines. Bar = 2.5 cm. (**b**) The rosette leaves from the plants of *bzr1-1D*, Col-0 and GmBZL2*-GFP transgenic lines from the top to the bottom. Bar = 2 cm. (**c**) The siliques of GmBZL2*-GFP transgenic lines and Col-0. Bar = 1 cm. (**d**) The 7-week-old seedlings of Col-0, *bzr1-1D* and the GmBZL2*-GFP transgenic lines. Bar = 5 cm. The kink inflorescence stem in the red box is the typical *bzr1-1D* like phenotype. (**e**) The transparent siliques of Col-0, *bzr1-1D*, and GmBZL2*-GFP trangenic lines. Bar = 4 mm. (**f**) Statistical analysis of seed number per silique from the 3^rd^ to 13^th^ silique on the primary stem (a total of 5 plants from each line) of wild type, *bzr1-1D* and transgenic lines. The student *t* test was used to analyze the significant differences among wild type, *bzr1-1D*, and independent GmBZL2*-GFP transgenic lines (***p < 0.001). (**g**) The 6-day-old seedling of Col-0 without BRZ treatment; the Col-0 and GmBZL2*-GFP transgenic lines grow on the 1/2MS medium with BRZ in dark. Bar = 1 cm. (**h**) Statistical analysis of 6-day-old seedling hypocotyl length of wild type (with and without BRZ treatment), *bzr1-1D* and GmBZL2*-GFP transgenic lines with BRZ treatment in dark (a total of 15 plants from each line). The student *t* test was used to analyze the significant differences between wild type and *bzr1-1D*, GmBZL2*-GFP transgenic lines with BRZ treatment (***p < 0.001). (**i**) The expression level of *CPD* and *DWF4* in 10-day-old seedlings of Col-0, *bzr1-1D*, and GmBZL2*-GFP transgenic lines.

Besides, we also analyzed another overexpression transgenic lines, pGmBZL2::GmBZL2*-GFP (the lines mentioned above for GmBZL2 activity detection). GmBZL2*-GFP lines showed enhanced phenotypes than both GmBZL2*-GUS lines and *bzr1-1D*. The leaves behaved severely curly than *bzr1-1D* (Fig. 5a,b). Apart from the leaves, the siliques are shrinking with bending pedicles compared with the wild type (Fig. 5c). The inflorescence stems of transgenic lines also typically kink toward to the axillary branches or leaves which mimics *bzr1-1D*^45^ (Fig. 5d). For the seed number, line 1 has less seed set than the wild type and *bzr1-1D*, and there were no significant differences in line 5 and line 6 compared with Col-0 (Fig. 5e,f). The ratio of dephosphorylated/phosphorylated GmBZL2 in line 1 was higher than line 6 (Supplementary Fig. S5e), which means BR signaling in line 1 was higher than line 6. We deduced that the decreased seed set in line 1 was a negative feedback of the BR signaling because of the high activity of GmBZL2 (detailed description in discussion). The expression levels of *CPD* and *DWF4* were lower in GmBZL2*-GFP transgenic lines, which demonstrates stronger BR signaling in GmBZL2*-GFP plants (Fig. 5i). All these results further illustrate the function and the regulatory mechanism of GmBZL2 would be conserved with AtBZR1.

We also treated GmBZL2*-GFP transgenic lines with BRZ in dark. The results suggested the hypocotyls of these transgenic lines were longer than both wild type and *bzr1-1D* seedling with BRZ treatment (but still shorter than Col-0 without BRZ treatment in Fig. 5g,h). Furthermore, we also treated the GmBZL2*-GFP lines with BR in light. The hypocotyl lengths of transgenic lines were not only longer than that of wild type, but also *bzr1-1D* (Supplementary Fig. S6b) under BR treatment. All these results reveal BR signaling in GmBZL2*-GFP transgenic lines are stronger than GmBZL2*-GUS transgenic lines and *bzr1-1D*, which consists to severely curly leaves and kink inflorescence stem phenotypes; as well as the feedback phenotypes of small rosette leaves and less seeds per silique.

### GmBZL2* overexpression partially rescues *bri1-5* phenotypes

**Figure 6:**
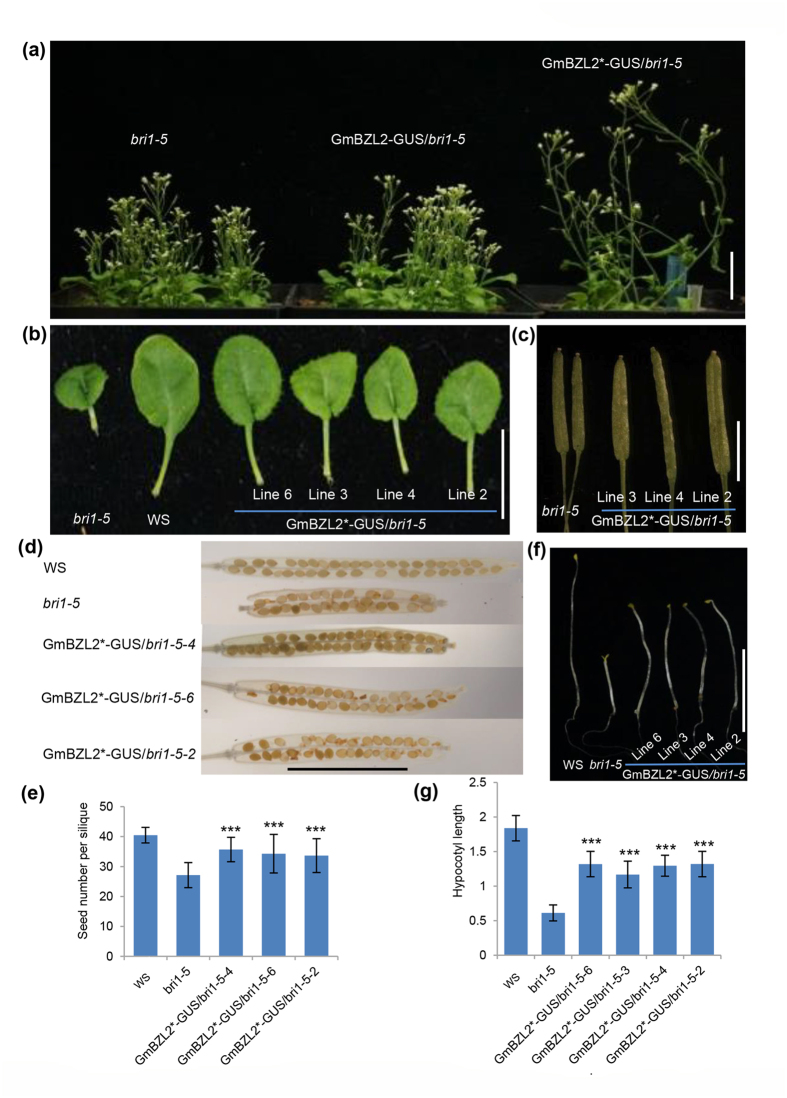
GmBZL2* partially rescues the BR-deficient phenotype of *bri1-5*. (**a**) The overview of 6-week-old plants of *bri1-5*, GmBZL2-GUS/*bri1-5* and GmBZL2*-GUS/*bri1-5* transgenic lines. Bar = 3 cm. (**b**) The petioles of *bri1-5*, wild type (WS) and GmBZL2*-GUS/*bri1-5* transgenic lines. Bar = 1 cm. (**c**) The siliques of *bri1-5* and the GmBZL2*-GUS/*bri1-5* lines. Bar = 0.5 cm. The student *t* test was performed between *bri1-5* and the transgenic lines (***P < 0.001). (**d**) Seed number per silique of WS, *bri1-5* and GmBZL2*-GUS/*bri1-5* transgenic lines. Bar = 0.5 cm. (**e**) Statistical analysis of seed number from the 3^rd^ to 13^th^ silique on the primary stem (a total of 5 plants from each line) of wild type, *bri1-5* and transgenic lines. The student *t* test was used to analyze the significant differences between *bri1-5* and GmBZL2*-GUS/*bri1-5* transgenic lines (***p < 0.001). (**f**) The 6-day-old seedling of WS*, bri1-5* and GmBZL2*-GUS/*bri1-5* transgenic lines grown in dark condition. Bar = 1 cm. (**g**) Statistical analysis of the 6-day-old seedling hypocotyl length of WS, *bri1-5* and GmBZL2*-GUS/*bri1-5* transgenic lines in dark (a total of 15 plants from each line). The student *t* test was used to analyze the significant differences between *bri1-5* and GmBZL2*-GUS/*bri1-5* transgenic lines (***p < 0.001).

Arabidopsis BR-insensitive mutant *bri1-5* shows phenotypes of dwarf, ground and dark green leaves, short petiole and less seeds per silique^2^,^30^. To confirm the conservative function of *GmBZL2*, we transformed GmBZL2*-GUS into *bri1-5* mutant and found that overexpression of *GmBZL2* could rescue *bri1-5* phenotypes. The plant height, petiole and silique length of transgenic lines of GmBZL2*-GUS/*bri1-5* (pGmBZL2::GmPZL2*-GUS/*bri1-5*) all increased (Fig. 6a–c). However, when we introduced GmBZL2-GUS to *bri1-5*, we did not observe significant difference in petiole length, plant height and seed number, compared with *bri1-5* (Fig. 6a). We also found GmBZL2* could improve the seed number per silique in *bri1-5*. Statistic results showed GmBZL2*-GUS/*bri1-5* transgenic lines had higher seed set than *bri1-5.* The average seed number per silique of *bri1-5* was 24.7, and the seed number per silique of independent transgenic lines were 35.7, 34.3, 33.7 in line 4, line 5, line 2, respectively (the average seed number per silique of WS was 40.5, Fig. 6d,e). The hypocotyl length of GmBZL2*-GUS/*bri1-5* was longer than *bri1-5* (Fig. 6f,g) in dark, indicating BR signaling has rescued in these transgenic lines. All these results illustrate that overexpression of GmBZL2 could rescue the BR-insensitivity phenotype of *bri1-5* mutant, like *bzr1-1D*, thus demonstrating that the conservation of BR signaling regulation of plant growth and development between soybean and Arabidopsis.

## Discussion

AtBZR1 (as well as AtBZR2/BES1) is the crucial transcription factor of BR signal transduction pathway. The gain-of-function mutant of AtBZR1, *bzr1-1D*, shows enhanced BR signaling, such as etiolation in dark (long hypocotyl) under BRZ treatment, longer petiole, kink inflorescence stem, larger flower and silique, more ovules per flower and more seeds per silique. When BR signaling is too high in some severe *bzr1-1D* plants, causing phenotypes like severely curly rosette leaves, weak growth, smaller flower and shorter silique, severely kink inflorescence stem and BRZ insensitivity in dark^8^. Too high *AtBZR1* transcription level doesn’t cause significant BR-signal-enhanced phenotypes^8^, because AtBZR1 activity is not influenced by transcription level of *AtBZR1*, but depends on its dephosphorization by BIN2. Besides, BR signaling would be down-regulated in feedback through inhibiting BR biosynthesis genes expression by AtBZR1.

The point mutation in P216L site of GmBZL2, which is conserved with P234L site in AtBZR1, leads to obvious phenotypes of enhanced BR signaling when the mutated GmBZL2 overexpressed in Arabidopsis (GmBZL2*-GUS, Fig. 4a–c), such as etiolation in dark (long hypocotyl) under BRZ treatment, longer petiole, larger rosette leaves and more seeds per silique. These phenotypes of transgenic lines are similar to AtBZR1 gain-of-function mutant *bzr1-1D*. Line 4 of GmBZL2*-GUS shows longest hypocotyl in dark under BRZ treatment among the 4 transgenic lines, even longer hypocotyl than *bzr1-1D*. And the expression inhibition of *CPD* and *DWF4* also reflects much enhanced BR signaling in line 4 (Fig. 4f–h), indicating that line 4 may have higher BR signaling than WT even *bzr1-1D* and it is high possibility to induce feedback inhibition of plant growth. About the rosette leaves and the seed number per silique, line 4 has smaller rosette leaves, shorter siliques and less seed number per silique than WT, indicating the feedback regulation of BR signaling and plant growth in line 4. The GUS stain results of seedlings in transgenic lines are consistent to the conclusion that line 4 has higher GmBZL2 activity and BR signaling than line 1 and line 7 (Supplementary Fig. S5a–d), further suggesting that line 4 has high BR signaling and leads to feedback inhibition of plant growth. Line 9 has similar GUS activity and rosette leaves size with line 4, but weak enhanced seed number per silique than WT, maybe because that the slight different strength of feedback inhibition determined by individual plant. But the difference between line 4 and line 1/7 is very clear.

GmBZL2*-GFP has more severe phenotypes resembling severe *bzr1-1D* plants, especially curly leaves and kink inflorescence stem (Fig. 5a–d). As well as the silique length and seed number per silique is lower or similar compared with WT, which is consistent to severe *bzr1-1D*. Among three independent transgenic lines, the ratio of dephosphrylated/phosphorylated GmBZL2 in line 1 is slightly higher than line 5 and line 6 (Supplementary Fig. S5e). It means BR signaling in line 1 is higher, which is consistent to the reduced seed set in line 1. The different phenotypes of GmBZL2*-GUS and GmBZL2*-GFP transgenic lines depends on whether enhance BR signaling induces feedback inhibition of BR synthesis and plant growth. Both fused GmBZL2 express in different transgenic lines with GUS or GFP tag, but GUS is much larger than GFP, which will lead to different efficiency in targeting and regulating downstream genes. It will be the reason for the different phenotypes of GmBZL2*-GUS and GmBZL2*-GFP transgenic lines.

BR signal transduction pathway has been well studied in Arabidopsis. AtBZR1 is the important BR-induced transcription factor which regulates downstream genes expression and feedback to inhibit BR biosynthesis. AtBZR1 activity depends on the ratio of dephosphorylated/phosphorylated AtBZR1. BR induces AtBZR1 activity by trigging its accumulation, dephosphorylation and re-localization to nuclear. The BR signal transduction pathway in soybean still remains unclear before. The identification and functional characterization of *GmBZL2* suggested that there would be conserved main regulator and mechanism of BR signal transduction pathway in soybean. GmBZL2* could partially rescue the phenotype of *bri1-5*, the insensitive mutant in BR pathway, indicating enhanced BR signaling by increasing GmBZL2 activity could complement the reduced BR signaling in *bri1-5*, similar to Arabidopsis BR enhanced mutant *bzr1-1D*. These results of genetic experiment demonstrate that soybean BR signal transduction pathway would be conserved with Arabidopsis.

The average seed number per silique is around 50 in Arabidopsis Col-0 ecotype. Seed number per silique determined by ovule number, male fertility, fertilization and zygote development. As the precursor of seed, ovule number determines the maximal possibility of seed number per silique. Our publication demonstrated that BR positively regulated ovule number by influencing ovule early development related gene expression by AtBZR1^30^. In this work, we also illustrated that GmBZL2* increased BR signaling and seed number per silique in Arabidopsis. We could hypothesize that GmBZL2* can increase seed number per pod in soybean, as similar as it functioned in Arabidopsis, since the enhanced GmBZL2 activity promoted BR signaling. Also similar to Arabidopsis, the BR signaling cannot be too high since it will induce the feedback inhibition of BR biosynthesis and lead to BR deficiency and reduced plant growth and seed yield. How to control BR signaling in suitable level is a challenge.

Although ovule grows up from parietal placenta (development from carpel margin meristem) in Arabidopsis and from central placenta in soybean; the possible BR regulation might be similar. Soybean has hundreds of pods and the average seed number per pod is around 3 (valued from different cultivars), suggesting the increase of seed number per pod would contribute enormously in soybean seed yield. How AtBZR1 and GmBZL2 work in different placenta pattern and play similar role in ovule identity and initiation to regulate seed set is worth investigating in future.

## Methods

### Plant materials, growth conditions and transformation procedure

In this study the wild type control is Arabidopsis ecotype Columbia-0 (Col-0) and soybean Zhongdou32. The BR-deficient mutant *bri1-5* in WS ecotype was transformed for the BR signaling rescue experiment. The tobacco *Nicotiana benthamiana* was used for BR induced nuclear localization and dephosphorylation of GmBZL2. All the plants were grown in the greenhouse where the temperature of 22 °C under 16 hr light and 8 hr dark.

Agrobacterium-mediated transformation was performed with floral dip method. The positive clones were cultured in YEP medium (peptone 10 g/L, yeast extract 10 g/L, NaCl 5 g/L, pH 7.2) with 50 mg/L rifampicin and 50 mg/L kanamycin for 10 hr at 28 °C, and the Agrobacterium was collected and diluted with 5% sucrose solution containing 0.02% (v/v) Silwet L-77 (SL77080596, GE) to OD_600_ between 0.8–1.0. The Arabidopsis inflorescences were dipped into the mixture buffer, sealed and kept in dark overnight.

### Phylogenetic analysis and exon-intron structure determination

The protein sequences and their corresponding CDS sequences of *BZR1*-like genes from representative whole genome-sequence species were retrieved by BLAST searches against Phytozome (http://phytozome.jgi.doe.gov), with an E-value cut-off of 10^5^. Protein sequences were aligned with Probalign^46^, and then manually adjusted using GeneDoc (version 2.6) software (Pittsburgh Supercomputing Center; http://www.psc.edu/biomed/genedoc/). The corresponding CDS alignment was generated using the PAL2NAL program (http://www.bork.embl.de/pal2nal/). Only the sites with column scores higher-than 6, which were estimated by CLUSTALX 1.83^47^, were used for phylogenetic analysis. Maximum-likelihood (ML) and neighbor-joining (NJ) trees were constructed with PhyML (version 2.4)^48^ and MEGA 6.0^49^, respectively. For the ML analysis, the GTR + I + Γ model was applied, and a BIONJ tree was used as a starting point for ML searches. For the NJ analysis, default parameters with pairwise deletion were selected. Bootstrap analyses were performed with 100 replicates in both ML and NJ analyses. Exon-intron structure diagrams for each gene were drawn in Gene Structure Display Server (http://gsds.cbi.pku.edu.cn/)^50^ based on the annotation information, which was retrieved from Phytozome.

For the alignment and the unrooted tree construction of BZR1-like genes in Arabidopsis and *Glycine max*, the software DNAMAN was used. The conservative domain was obtained from the SAMRT website(http://smart.embl-heidelberg.de/).

### Construct of GmBZL2-GUS, GmBZL2*-GUS, GmBZL2-GFP and GmBZL2*-GFP

A 2860 bp gDNA of *GmBZL2* containing 932 bp promoter was amplified with Q5™ High-Fidelity DNA Polymerase (M0491; New England Biolabs), digested with *Eco*RI and *Bgl*II (FD0375, FD0083; Thermo Fisher Scientific) and then cloned into pCAMBIA1301 (CAMBIA, Canberra, ACT, Australia) for the vector GmBZL2-GUS. The specific primers for the gDNA were proGmBZL2-*Eco*R I-F (5′-CCGGAATTCGTAATGTTGGTGATGAAGGATG-3′) and proGmBZL2-CA-*Bgl* II-R (5′-GGAAGATCTACACTCCGCACCTTCCCACTT-3′). To construct GmBZL2*-GUS, primer pairs proGmBZL2-*Eco*R I-F/M-GmBZL2-R(5′-GGAATAGTGGGCAGAGTGTA-3′) and M-GmBZL2-F (5′-TACACTCTGCCCACTATTCC-3′)/proGmBZL2-CA-*Bgl* II-R were used to amplified the first and the second half of the 2860 bp gDNA. Two products were purified and mixed equally for the overlapping PCR with primers proGmBZL2-*Eco*R I-F and proGmBZL2-CA-*Bgl* II-R. Then the full length GmBZL2* with a site mutation was cloned into pCAMBIA1301. Meanwhile, the same gDNA fragment with a site mutation was also cloned into pCAMBIA1302 (CAMBIA, Canberra, ACT, Australia) for the construct GmBZL2*-GFP. The construct procedure of GmBZL2-GFP was same to GmBZL2-GUS but into pCAMBIA1302 vector.

### QRT-PCR assay and promoter fusion analysis

The total RNA from 10-day-old seedlings was extracted with TRIZOL (T9424, Sigma-Aldrich). 1 μg RNA was reverse transcribed with RT reagent Kit with gDNA Eraser (RR047A, TAKARA) into cDNA. The qRT-PCR was carried out with SYBR Green Realtime PCR Master Mix (QPK-201T,TOYOBO) in a 20 μl volume on a Bio-Rad Real-Time PCR Detection System. The specific primers of *GmBZL2* were GmBZL2-RT-F (5′-CGAGCTCAGGGGAACTTCAA-3′) and GmBZL2-RT-R (5′-TTGGGCTGGGAAATGACGAA-3′). The primers for *CPD* were CPD RT-F (5′-CATGGAAGAAGCCAAAAAGATAACG-3′) and CPD RT-R (5′-CTTTGCGGTAAGTGGTGGAGA-3′). The primers for *DWF4* were DWF4 RT-F (5′-CATGTCTCCAAGTATGGTAAGATAT-3′) and DWF4 RT-R (5′-ATTTCCCAAGAATCCCACCTATACT-3′). The inner reference gene was *ACTIN* and the primers were 5′-CCGGTATTGTGCTCGATTCTG-3′ and 5′-TTCCCGTTCTGCGGTAGTGG-3′. The procedure was 40 cycles of 94 °C for 10 s, 60 °C for 15 s, 72 °C for 20 s.

The inflorescence of GmBZL2-GUS lines were dissected and vacuumized in the GUS staining solution at 37 °C for 30 min, kept at 37 °C overnight, then the chlorophyll was eluted with 75% alcohol twice. The GUS staining tissues were observed with stereomicroscope Leica S8APO and taken photographed with digital camera Leica DFC450.

### Characterization of the GmBZL2*-GUS/GFP overexpression lines

The phenotype of 4-week-old plants and leaf shape of GmBZL2*-GUS/GFP were photographed with a Canon EOS60D digital camera. The 10-day-old siliques from different lines were observed on a stereomicroscope Leica S8APO and photographed with Leica DFC450. For seed number counting the 10-day-old siliques were cleared with Hoyer’s solution (chloral hydrate: glycerol: water = 8:1:3, w/v/v) at 4 °C overnight, and counted the seed number with Leica S8APO.

### BRZ and BR treatment

The seeds were sterilized and sown on 1/2MS medium containing 2 μM BRZ or 10 nM BR, vernalized at 4 °C for 48 hr and grown vertically at 22 °C in dark or 16 hr light and 8 hr dark. The 6-day-old were photographed with Leica DFC450.

### Treatment with chemicals for protein analysis

10-day-old seedlings grown on 1/2MS medium were removed from petri dishes and submerged into liquid 1/2MS medium with or without LiCl for 3 hr. The protein was extracted with PEB buffer for Western blot. The GmBZL2-GFP and GmBZL2*-GFP transgenic plants were grown in 1/2MS medium with or without 5 μM bikinin in the growth chamber at 22 °C under long-day condition (16 hr light/8 hr dark). Total protein was extracted from 7-day-old seedlings for Western blot.

### Nuclear localization and dephosphorylation of GmBZL2 with BR inducement

The 35S::GmBZL2-GFP vector was constructed with Gateway Recombinational Cloning Technology. The 933 bp CDS of *GmBZL2* without stop codon was amplified from the cDNA of soybean flower with primers GmBZL2-F (5′-GGGGACAAGTTTGTACAAAAAAGCAGGCTTCATGGTCGACGACGGAGCAA-3′) and GmBZL2-R (5′-GGGGACCACTTTGTACAAGAAAGCTGGGTTACTCCGCACCTTCCCACTT-3′), cloned into intermediate vector pDONR207 with BP reaction (11789-020; Life Technologies) for homologous recombination, then the *GmBZL2* fragment was exchanged into the modulated destination vector pEarlyGate103 with LR recombinant reaction (11791-020; Life Technologies).

The positive clone was introduced into *Agrobacterium tumefaciens* GV3101, cultured and transformed with injection into four-week-old tobacco leaves, and then the leaves were cut down and immersed into 10 μM eBL elution buffer for 8 hr after 48 hr in dark. The epidermis layer of tobacco leaf was teared out, immersed into water, and the GFP signal was excited at a 488 nm wavelength and the emission was collected at 518 nm with Olympus laser confocal microscopy FluoView™ FV1000.

For Western blot, the same transgenic tobacco leaves were treated with 100 μM eBL for 10 hr. The protein was extracted with PEB buffer (50 mM Tris-HCl pH = 7.5, 1 mM EDTA pH = 7.5, 150 mM NaCl, 5% glycerol, 0.5% Trion-X-100), and the protein concentration was determined with Thermo Scientific NanoDrop 2000/2000 C. 20 mg protein was loaded and separated on a 12% SDS-PAGE, transferred to nitrocellulose membrane at 100 mA for 1 hr. The membranes were blocked in PEB buffer with 1% BSA for 1 hr at room temperature, probed with anti-GFP antibody (sc-9996, Senta Cruz Biotechnology) and incubated with alkaline phosphatase-conjugated secondary antibody (sc-2008, Senta Cruz Biotechnology). After adding the substrate BCIP/NBT (002209, Invitrogen) for 15 min, the reaction was stopped for belt observation.

## Additional Information

**How to cite this article**: Zhang, Y. *et al.* Functional characterization of *GmBZL2* (*AtBZR1* like gene) reveals the conserved BR signaling regulation in *Glycine max.* *Sci. Rep.* **6**, 31134; doi: 10.1038/srep31134 (2016).

## Supplementary Material

Supplementary Information
