# Supplementary material for: Functional characterization of GmBZL2 (AtBZR1 like gene) reveals the conserved BR signaling regulation in Glycine max
Source: Sci Rep. 2016 Aug 8;6:31134. doi: 10.1038/srep31134 (PMC4976319; doi:10.1038/srep31134)
Supplement: Supplementary Information [file srep31134-s1.pdf]

Functional characterization of *GmBZL2* (*AtBZR1* like gene) reveals the conserved BR signaling regulation in *Glycine max*

Yu Zhang[1,5,+], Yan-Jie Zhang[2,+], Bao-Jun Yang[3], Xian-Xian Yu[4,5], Dun Wang[4], Song-Hao Zu[2], Hong-Wei Xue[3] and Wen-Hui Lin[2,*]

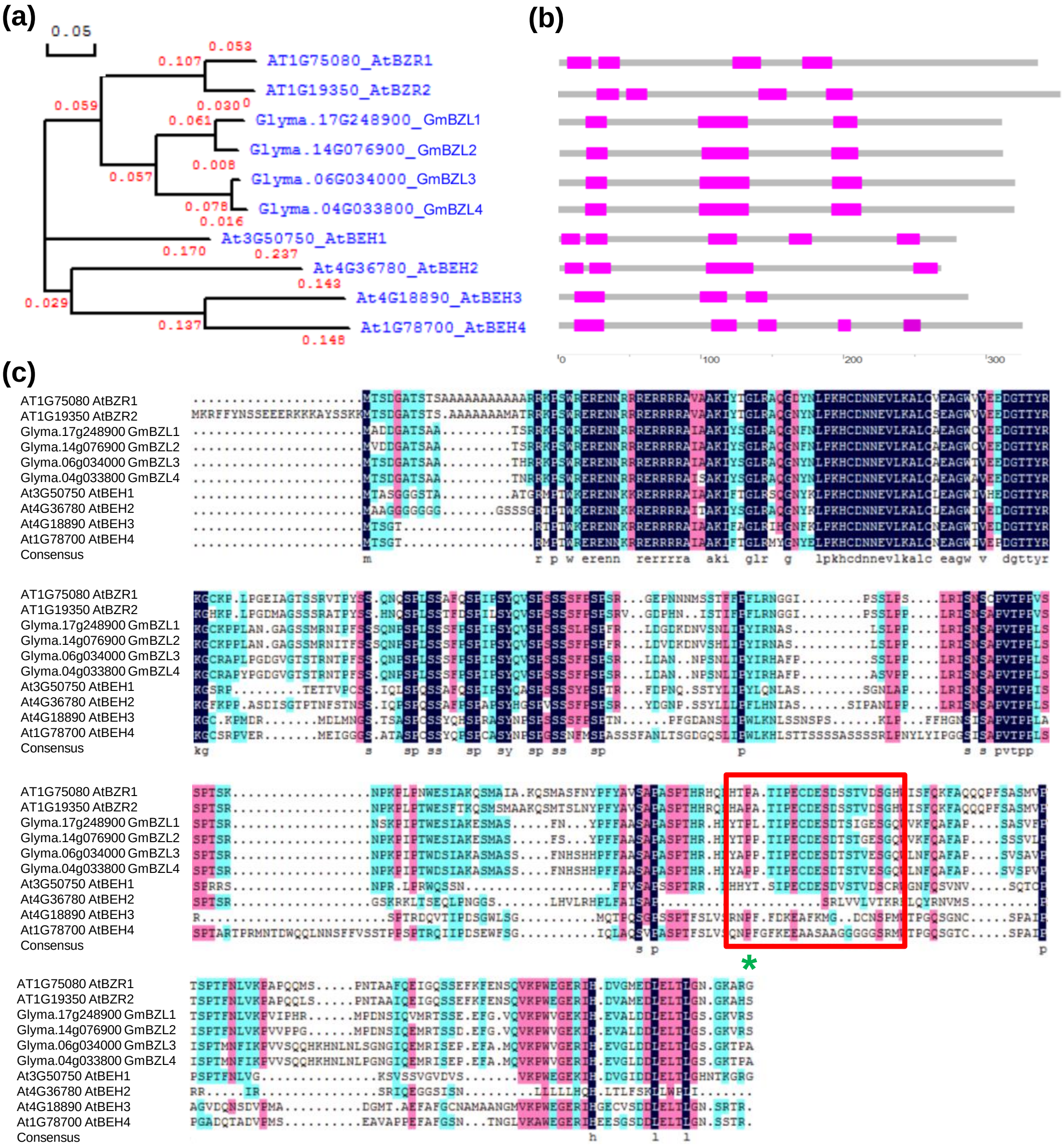

Supplementary Figure S1. Phylogenetic analysis of *BZR1*-like genes in *Glycine max* and Arabidopsis. (**a**) The unrooted tree of BZR1-like genes in *Glycine max* and Arabidopsis. (**b**) The schematic representation of conserved domain of BZR1-like genes in *Glycine max* and Arabidopsis. (**c**) The alignment of the amino acid sequences of AtBZR1, AtBZR2(AtBES1), GmBZL1, GmBZL2, GmBZL3, GmBZL4, AtBEH1, AtBEH2, AtBEH3 and AtBEH4. The PEST domains were shown in the red rectangle. The conserved proline sites (P234L mutated in *bzr1-1D*) or putative conserved proline sites P were marked with green asterisk.

(a) Expression Quantification of *GmBZL1*

Expression Quantification
There are 26 expression records

Experiment Name	Experiment Group	FPKM
Flower	Tissue Sample	7.87742
Flower.open	GeneAtlas Tissue Sample	5.535
Flower.unopen	GeneAtlas Tissue Sample	4.589
Lateral root.standard	GeneAtlas Tissue Sample	5.113
Leaf.ammonia	GeneAtlas Tissue Sample	4.561
Leaf.nitrate	GeneAtlas Tissue Sample	4.349
Leaf.standard	GeneAtlas Tissue Sample	4.009
Leaf.symbiotic condition	GeneAtlas Tissue Sample	4.46
Leaf.urea	GeneAtlas Tissue Sample	4.812
Leaves	Tissue Sample	5.74484
Nodules	Tissue Sample	6.52086
Nodules.symbiotic condition	GeneAtlas Tissue Sample	0.666
Pod	Tissue Sample	8.55525
Root	Tissue Sample	8.89899
Root.ammonia	GeneAtlas Tissue Sample	1.336
Root Hairs	Tissue Sample	12.6793
Root.nitrate	GeneAtlas Tissue Sample	1.346
Root.standard	GeneAtlas Tissue Sample	0.926
Root.symbiotic condition	GeneAtlas Tissue Sample	1.075
Root tip.standard	GeneAtlas Tissue Sample	5.7
Root.urea	GeneAtlas Tissue Sample	1.052
Seed	Tissue Sample	7.22565
Shoot Apical Meristem	Tissue Sample	15.1282
Shoot tip.standard	GeneAtlas Tissue Sample	8.934
Stem	Tissue Sample	14.3446
Stem.standard	GeneAtlas Tissue Sample	8.558

(b) Expression Quantification of *GmBZL2*

Expression Quantification
There are 26 expression records

Experiment Name	Experiment Group	FPKM
Flower	Tissue Sample	17.8006
Flower.open	GeneAtlas Tissue Sample	8.172
Flower.unopen	GeneAtlas Tissue Sample	3.068
Lateral root.standard	GeneAtlas Tissue Sample	2.646
Leaf.ammonia	GeneAtlas Tissue Sample	2.898
Leaf.nitrate	GeneAtlas Tissue Sample	2.866
Leaf.standard	GeneAtlas Tissue Sample	2.708
Leaf.symbiotic condition	GeneAtlas Tissue Sample	3.082
Leaf.urea	GeneAtlas Tissue Sample	2.905
Leaves	Tissue Sample	4.40739
Nodules	Tissue Sample	3.66962
Nodules.symbiotic condition	GeneAtlas Tissue Sample	0.477
Pod	Tissue Sample	4.74082
Root	Tissue Sample	8.26335
Root.ammonia	GeneAtlas Tissue Sample	1.649
Root Hairs	Tissue Sample	5.92523
Root.nitrate	GeneAtlas Tissue Sample	1.059
Root.standard	GeneAtlas Tissue Sample	0.848
Root.symbiotic condition	GeneAtlas Tissue Sample	1.337
Root tip.standard	GeneAtlas Tissue Sample	2.216
Root.urea	GeneAtlas Tissue Sample	1.075
Seed	Tissue Sample	2.154
Shoot Apical Meristem	Tissue Sample	5.41563
Shoot tip.standard	GeneAtlas Tissue Sample	3.651
Stem	Tissue Sample	7.47842
Stem.standard	GeneAtlas Tissue Sample	4.247

(c) Expression Quantification of *GmBZL3*

Expression Quantification
There are 26 expression records

Experiment Name	Experiment Group	FPKM
Flower	Tissue Sample	4.10834
Flower.open	GeneAtlas Tissue Sample	4.88
Flower.unopen	GeneAtlas Tissue Sample	6.707
Lateral root.standard	GeneAtlas Tissue Sample	2.533
Leaf.ammonia	GeneAtlas Tissue Sample	7.718
Leaf.nitrate	GeneAtlas Tissue Sample	6.356
Leaf.standard	GeneAtlas Tissue Sample	4.447
Leaf.symbiotic condition	GeneAtlas Tissue Sample	6.956
Leaf.urea	GeneAtlas Tissue Sample	5.654
Leaves	Tissue Sample	0.584785
Nodules	Tissue Sample	5.3586
Nodules.symbiotic condition	GeneAtlas Tissue Sample	0.063
Pod	Tissue Sample	27.7801
Root	Tissue Sample	3.56679
Root.ammonia	GeneAtlas Tissue Sample	0.44
Root Hairs	Tissue Sample	9.81962
Root.nitrate	GeneAtlas Tissue Sample	0.567
Root.standard	GeneAtlas Tissue Sample	0.214
Root.symbiotic condition	GeneAtlas Tissue Sample	0.249
Root tip.standard	GeneAtlas Tissue Sample	3.052
Root.urea	GeneAtlas Tissue Sample	0.44
Seed	Tissue Sample	9.5602
Shoot Apical Meristem	Tissue Sample	16.9477
Shoot tip.standard	GeneAtlas Tissue Sample	7.881
Stem	Tissue Sample	11.5993
Stem.standard	GeneAtlas Tissue Sample	8.695

(d) Expression Quantification of *GmBZL4*

Expression Quantification
There are 26 expression records

Experiment Name	Experiment Group	FPKM
Flower	Tissue Sample	4.48286
Flower.open	GeneAtlas Tissue Sample	6.344
Flower.unopen	GeneAtlas Tissue Sample	6.478
Lateral root.standard	GeneAtlas Tissue Sample	3.519
Leaf.ammonia	GeneAtlas Tissue Sample	4.419
Leaf.nitrate	GeneAtlas Tissue Sample	4.523
Leaf.standard	GeneAtlas Tissue Sample	5.26
Leaf.symbiotic condition	GeneAtlas Tissue Sample	3.596
Leaf.urea	GeneAtlas Tissue Sample	4.589
Leaves	Tissue Sample	2.3987
Nodules	Tissue Sample	5.59032
Nodules.symbiotic condition	GeneAtlas Tissue Sample	0.689
Pod	Tissue Sample	6.81391
Root	Tissue Sample	3.86044
Root.ammonia	GeneAtlas Tissue Sample	0.553
Root Hairs	Tissue Sample	8.47919
Root.nitrate	GeneAtlas Tissue Sample	1.978
Root.standard	GeneAtlas Tissue Sample	1.4
Root.symbiotic condition	GeneAtlas Tissue Sample	0.438
Root tip.standard	GeneAtlas Tissue Sample	4.815
Root.urea	GeneAtlas Tissue Sample	0.996
Seed	Tissue Sample	7.70541
Shoot Apical Meristem	Tissue Sample	10.6149
Shoot tip.standard	GeneAtlas Tissue Sample	9.415
Stem	Tissue Sample	6.45977
Stem.standard	GeneAtlas Tissue Sample	5.481

Supplementary Figure S2. Expression quantification of *GmBZL1/2/3/4* in *Glycine max* . Expression quantification of *GmBZL1* (a) (https://phytozome.jgi.doe.gov/phytomine/portal.do?externalid=PAC:30479951&class=gene), *GmBZL2* (b) (https://phytozome.jgi.doe.gov/phytomine/portal.do?externalid=PAC:30533621&class=gene), *GmBZL3* (c) (https://phytozome.jgi.doe.gov/phytomine/portal.do?externalid=PAC:30550683&class=gene), and *GmBZL4* (d) (https://phytozome.jgi.doe.gov/phytomine/portal.do?externalid=PAC:30488412&class=gene).

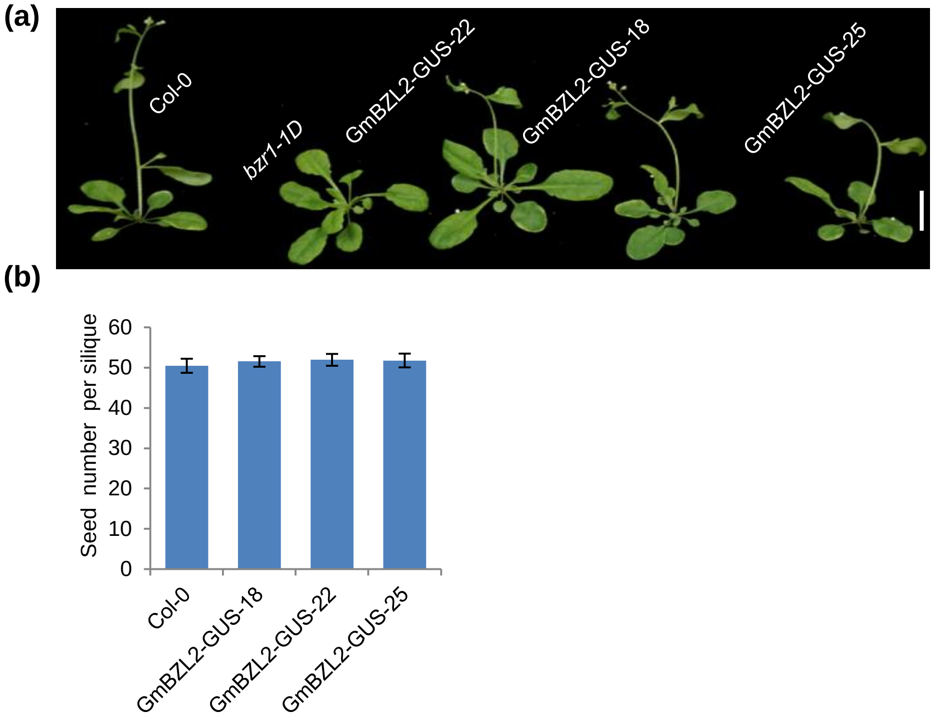

Supplementary Figure S3. The characterization of the GmBZL2-GUS lines. (**a**) The 6-week-old plants of Col-0, *bzr1-1D*, and GmBZL2-GUS transgenic lines. Bar=2 cm. (**b**) The seed number per silique in Col-0, *bzr1-1D*, and GmBZL2-GUS transgenic lines.

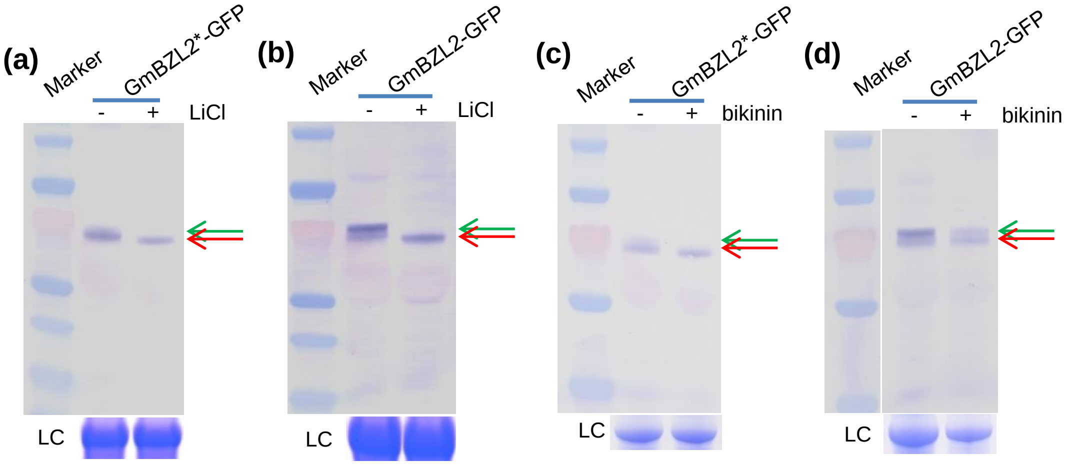

Supplementary Figure S4. LiCl and bikinin treatments increase the ratio of dephosphorylation/phosphorylation of GmBZL2 and GmBZL2*. Complete elimination of phosphorylated GmBZL2* by treatment with 100 mM LiCl in GmBZL2*-GFP transgenic line (**a**) and GmBZL2-GFP transgenic line (**b**). The ratio of dephosphorylation/phosphorylation was inceased in both GmBZL2*-GFP transgenic lines (**c**) and GmBZL2-GFP transgenic line (**d**) with 5 µM bikinin treatment**.** Ribosome bands were used as loading control. The green and red arrows indicate the phosphorylated and dephosphorylated proteins, respectively. The six protein marker belts are 130kD, 100 kD, 70 kD, 55 kD, 40 kD and 35 kD from top to bottom in (**a**) and (**b**), the five protein marker belts are 130kD, 100 kD, 70 kD, 55 kD and 35 kD from top to bottom in (**c**) and (**d**), LC indicates loading control.

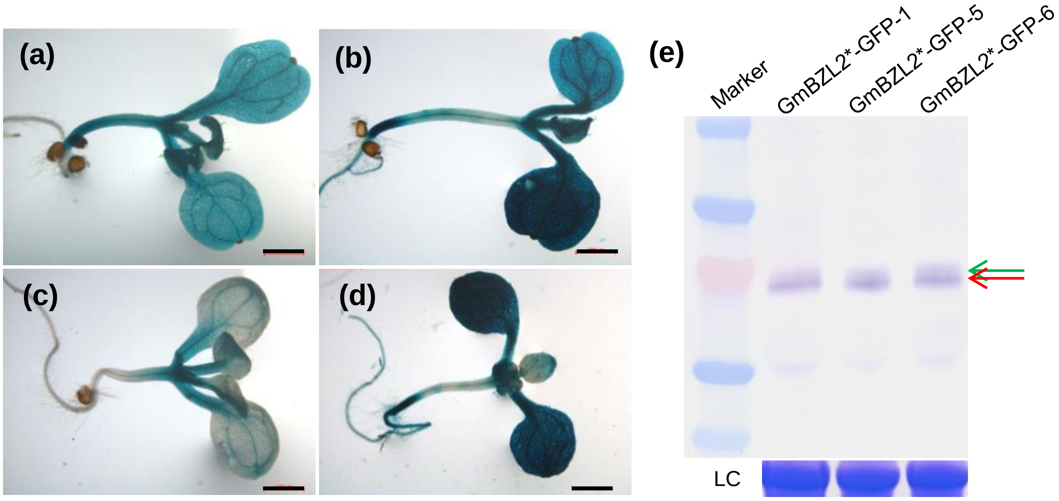

Supplementary Figure S5. Expression level of GmBZL2*. (**a-d**) GUS staining of 7-day-old seedling of GmBZL2*-GUS transgenic lines. Bar=1 mm. The expression level of GmBZL2* was higher in line 4 (**b**) and line 9 (**d**) than line 1 (**a**) and line 7 (**c**). (**e**) Western blot analysis of GmBZL2* protein level in GmBZL2*-GFP transgenic lines. The ratio of dephosphorylation/phosphorylation of GmBZL2* is higher in line 1 and line 5. The green and red arrows indicate the phosphorylated and dephosphorylated proteins, respectively. Ribosome bands were used as loading control. The five protein marker belts are 130kD, 100 kD, 70 kD, 55 kD and 40 kD from top to bottom, LC indicates loading control.

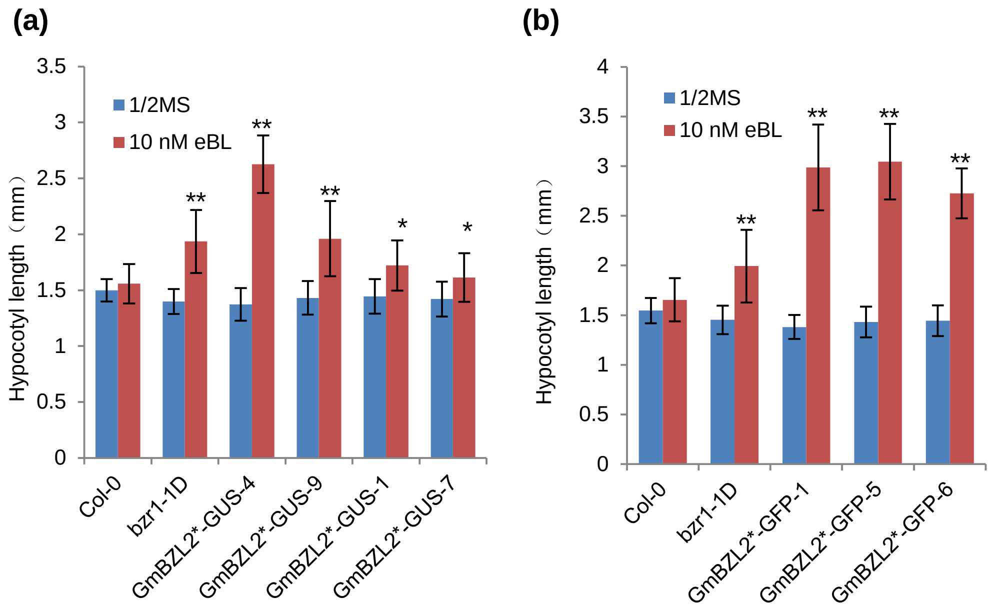

Supplementary Figure S6. GmBZL2* is hypersensitive to BR. (**a**) Hypocotyl lengths of 7-day-old seedlings of Col-0, *bzr1-1D* and GmBZL2*-GUS transgenic lines, which were all grown in 16 hr light and 8 hr dark on 1/2MS medium containing 0 or 10 nM eBL. The student *t* test was used to analyze the significant differences among wild type and *bzr1-1D*, GmBZL2*-GFP transgenic lines with BR treatment (**p < 0.01, *p < 0.05). (**b**) Hypocotyl lengths of 7-day-old seedlings of Col-0, *bzr1-1D* and GmBZL2*-GFP transgenic lines, which were all grown in 16 hr light and 8 hr dark on 1/2MS medium containing 0 or 10 nM eBL. The student *t* test was used to analyze the significant differences among wild type and *bzr1-1D*, GmBZL2*-GFP transgenic lines with BR treatment (**p < 0.01).
